# Predicting species establishment using absent species and functional neighborhoods

**DOI:** 10.1002/ece3.2804

**Published:** 2017-03-04

**Authors:** Jonathan A. Bennett, Meelis Pärtel

**Affiliations:** ^1^ Institute of Ecology and Earth SciencesUniversity of TartuTartuEstonia; ^2^ Present address: Department of BiologyUniversity of British Columbia – Okanagan CampusKelownaBCCanada

**Keywords:** alvar grasslands, community assembly, competitive hierarchies, dark diversity, Darwin's naturalization hypothesis, environmental filtering, filtered species pool, functional diversity, phylogenetic community ecology, weak competitor exclusion

## Abstract

Species establishment within a community depends on their interactions with the local environment and resident community. Such environmental and biotic filtering is frequently inferred from functional trait and phylogenetic patterns within communities; these patterns may also predict which additional species can establish. However, differentiating between environmental and biotic filtering can be challenging, which may complicate establishment predictions. Creating a habitat‐specific species pool by identifying which absent species within the region can establish in the focal habitat allows us to isolate biotic filtering by modeling dissimilarity between the observed and biotically excluded species able to pass environmental filters. Similarly, modeling the dissimilarity between the habitat‐specific species pool and the environmentally excluded species within the region can isolate local environmental filters. Combined, these models identify potentially successful phenotypes and why certain phenotypes were unsuccessful. Here, we present a framework that uses the functional dissimilarity among these groups in logistic models to predict establishment of additional species. This approach can use multivariate trait distances and phylogenetic information, but is most powerful when using individual traits and their interactions. It also requires an appropriate distance‐based dissimilarity measure, yet the two most commonly used indices, nearest neighbor (one species) and mean pairwise (all species) distances, may inaccurately predict establishment. By iteratively increasing the number of species used to measure dissimilarity, a functional neighborhood can be chosen that maximizes the detection of underlying trait patterns. We tested this framework using two seed addition experiments in calcareous grasslands. Although the functional neighborhood size that best fits the community's trait structure depended on the type of filtering considered, selecting these functional neighborhood sizes allowed our framework to predict up to 50% of the variation in actual establishment from seed. These results indicate that the proposed framework may be a powerful tool for studying and predicting species establishment.

## 1 Introduction

The establishment of species within a community is both a product and driver of community dynamics (Davis, Thompson, & Grime, 2005; Tilman, 2004). Consequently, quantifying the establishment potential of different species among habitats is a primary goal in community ecology. However, predicting establishment is complex; it depends on the availability of propagules, the match between the species’ environmental tolerances and the target habitat, and the interactions between the species and other organisms within that habitat (Meiners, Cadotte, Fridley, Pickett, & Walker, 2015; Seastedt & Pysek, 2011).

If a species has sufficient propagules reaching a site, establishment next depends on whether the species has the appropriate characteristics to cope with the local environment. This can be assessed by measuring the dissimilarity between the potential colonist and the resident community (Gallien, Carboni, & Munkemuller, 2014; Laughlin, Joshi, van Bodegom, Bastow, & Fulé, 2012; Moles, Gruber, & Bonser, 2008; Shipley, Vile, & Garnier, 2006; Thuiller et al., 2010). For a species to pass the environmental filters (environmental filtering; see Box 1 for definitions), the expectation is that the species should be similar to the resident community. The likelihood of passing the environmental filters can therefore be estimated by modeling the relationship between the species and the functional and phylogenetic structure of the community (e.g., Laughlin et al., 2012; Shipley et al., 2006; Warton, Shipley, & Hastie, 2015).

Box 1 **Definitions of terms used throughout the manuscript**

1

**Table:** 

Term	Definition
Regional species list	The complete inventory of species found within a given region, where the spatial extent of the region is delimited by dispersal distances of the species within the list. The size of the region, and thus the extent of the species list, can be adjusted to fit both short‐ and long‐term assumptions of dispersal distance by increasing spatial extent
Habitat‐specific species pool	The subset of the regional species list that possess the characteristics enabling them to colonize a given community
Dark diversity	The portion of the habitat‐specific species pool that is absent from a given community
Environmental filtering	The process by which species are excluded from a community based on whether they possess the traits required to inhabit a given environment (also known as abiotic filtering)
Biotic filtering	The process by which species are excluded from a community through interactions with other organisms (a.k.a. biotic resistance)
Limiting similarity	A mode of biotic filtering that occurs when two species are unable to coexist because they are too similar resulting in increased dispersion of trait values within the community
Weak phenotype exclusion	A mode of biotic filtering where all species that lack a particular set of traits are excluded through biological interactions resulting in trait clustering (a.k.a. weak competitor exclusion or competitive hierarchy)
Functional space	Multivariate ordination space based on the distribution of trait values among all species within the regional list. Can also be used for defining distances among species using standardized individual trait values
Functional neighborhood	The set of species in close proximity of a target species within functional space. The extent of the neighborhood can be defined in multiple ways
Functional distance	A measure of dissimilarity based on the distance between species in functional space

After passing the environmental filters, species must then pass biotic filters. Species can be successful in this regard if they are either dissimilar or similar to the resident community (de Bello et al., 2012; Grime, 2006; Mayfield & Levine, 2010). If establishing species are dissimilar to the resident community, they may occupy a distinct niche and avoid strong interactions (limiting similarity; MacArthur & Levins, 1967). If establishing species are similar to the resident community, they may share a competitively dominant phenotype that minimizes fitness differences with the resident biota and permits coexistence (weak competitor or phenotype exclusion; de Bello et al., 2012). Due to the complex relationship between similarity and biotic filtering, the signal of biotic interactions within communities can be more difficult to detect and their consequences for establishment more difficult to predict, especially in the face of strong environmental filtering (de Bello et al., 2012; Carboni et al., 2016; Gallien et al., 2014; Lessard et al., 2016; Spasojevic & Suding, 2012). Moreover, many biotic interactions can have equalizing effects on species coexistence; dissimilarity in competitive abilities can be offset if the inferior competitor is less affected by herbivory (Gross, Liancourt, Butters, Duncan, & Hulme, 2015; Heard & Sax, 2013). Consequently, whether potential colonists should be more or less similar to the resident community will depend on the nature of the biotic interactions in the community and how different traits affect those interactions (MacDougall, Gilbert, & Levine, 2009).

There are multiple current approaches to identifying and predicting the effects of biotic interactions on community assembly. By considering only species that can colonize the focal habitat (the habitat‐specific species pool), null models can identify the signal of biotic interactions, irrespective of whether biotic interactions lead to species being either more or less similar (de Bello et al., 2012; Chalmandrier et al., 2013; Lessard et al., 2016). However, null models do not allow predictions of establishment of new species into the community. Comparing potential colonists to the resident species at multiple spatial scales (i.e., local versus regional diversity) can identify both environmental and biotic filtering and may predict establishment (Carboni et al., 2016; Gallien et al., 2014; Lemoine et al., 2015). Such regression‐based approaches can also include interactions among traits, which may be critical for determining establishment success if biotic and environmental filters require the species to meet several criteria (Küster, Kühn, Bruelheide, & Klotz, 2008). However, none have done so to date. Regression‐based approaches have yet to use information on observed and absent species from within regional and habitat‐specific species pools to develop a priori predictions of the relationship between species characteristics and different community assembly processes. Such information could improve our understanding of the processes that limit establishment (Lewis, de Bello et al., 2017; Zenni & Nuñez, 2013). A regression approach that uses a species’ dissimilarity to both present and absent species, and defines the mechanism for those absences, may better predict which species can establish within a given site.

Using dissimilarity as a means of predicting establishment requires the choice of an appropriate distance metric (Carboni et al., 2016; Gallien et al., 2014). Trait and phylogenetic studies typically use some version of either nearest neighbor or mean pairwise distances among species to evaluate the role of dissimilarity in community assembly or species establishment (Gallien et al., 2014; Kraft & Ackerly, 2010; Petchey & Gaston, 2006; Thuiller et al., 2010; Webb, Ackerly, McPeek, & Donoghue, 2002). However, both nearest neighbor and mean pairwise distances make important assumptions about the relationship between dissimilarity and successful establishment. By including only the most similar species, nearest neighbor distances assume that the only important interaction is with that single other species. While interactions may be pairwise in some communities (Kelly, Bowler, Pybus, & Harvey, 2008), in most communities biotic interactions are diffuse (Mitchley, 1987) and the potential for multiple species to influence biotic resistance is high (White, Wilson, & Clarke, 2006). This suggests that mean pairwise distances may be a more appropriate measure of dissimilarity, consistent with recent simulation results (Gallien et al., 2014). However, mean pairwise distances assume that all co‐occurring species affect establishment success. This may not be true if exclusion occurs by strong competition driven by limiting similarity among a subset of the species. The number of species involved in these interactions could be anywhere between one and all species, so the number of species included in distance‐based dissimilarity indices should range between nearest neighbor and mean pairwise distances. We call this subset of species the functional neighborhood.

In this paper, we first introduce a framework for using the traits and phylogenetic relationships within the region and the local community to model community assembly and predict establishment. We then discuss the advantages of different dissimilarity measures, introducing a neighborhood approach to measuring functional dissimilarity. Finally, we demonstrate the application of the framework by predicting the establishment of species added as seed to two species‐rich calcareous grasslands in western Estonia (Zobel, Otsus, Liira, Moora, & Möls, 2000; Zobel, Otsus, Rünk, & Liira, 2005).

## 2 An Overview of the Framework

To use present and absent species to predict establishment within a specific community requires comparison of species characteristics across three levels of organization: the regional species list; the habitat‐specific species pool, and the locally observed community. This requires gathering data on both regional and local diversity and identifying an appropriate habitat‐specific species pool, as in null modeling approaches (de Bello et al., 2012; Chalmandrier et al., 2013; Lessard et al., 2016). Diversity at these three scales must then be combined with information on species’ functional traits or phylogenetic relationships. The species in the region, but absent from the habitat‐specific species pool (hereafter environmentally excluded species) can then be compared to the habitat‐specific species pool to define the set of characteristics required to pass the environmental filters. This relationship can be quantified using logistic regression with presence or absence in the habitat‐specific species pool as success or failure. Using a similar logistic regression approach, the characteristics of species absent from the local community but present in the habitat‐specific species pool (biotically excluded species or dark diversity; Pärtel, Szava‐Kovats, & Zobel, 2011) are compared to species observed within the community to identify whether the species within the community are more dissimilar (limiting similarity) or similar (weak phenotype exclusion) to each other than expected by chance. Using these two sets of logistic regressions, we can predict the probability that a new species will establish based on their similarity to the habitat‐specific species pool and locally observed species. Importantly, as a logistic modeling framework, this approach can use any measure of distance (e.g., multivariate trait distances, phylogenetic relationships, multiple individual traits) as well as interactions among distance measures.

### 2.1 Defining species pools

To use species presences and absences for predicting establishment, appropriate species pools must be defined. The regional species list should contain most species found within the region of interest and can be obtained through surveys, compiled data from the region, or from appropriate floras or faunas. The composition of species present within the local community can be measured using any number of community survey techniques. By contrast, one must estimate the remainder of the habitat‐specific species pool. Published species occupancy data from similar sites can be filtered by the regional species list. Alternatively, methods using dispersal probabilities, species co‐occurrences, and environmental tolerances can estimate membership in the habitat‐specific species pool (de Bello et al., 2012, 2016; Karger et al., 2016; Lessard et al., 2016; Lewis, Szava‐Kovats, Pärtel, & Isaac, 2016; Pärtel et al., 2011; Riibak et al., 2015). Categorizing absent species in this way splits the regional species list into absent species that cannot inhabit a site (environmentally excluded), absent species that can inhabit a site (biotically excluded), and locally observed species, allowing comparisons among these groups. However, the delineations of environmental and biotic exclusion are based on the realized niche, which could confuse these two processes (Kraft et al., 2015).

### 2.2 Environmental filtering

**Figure 1:**
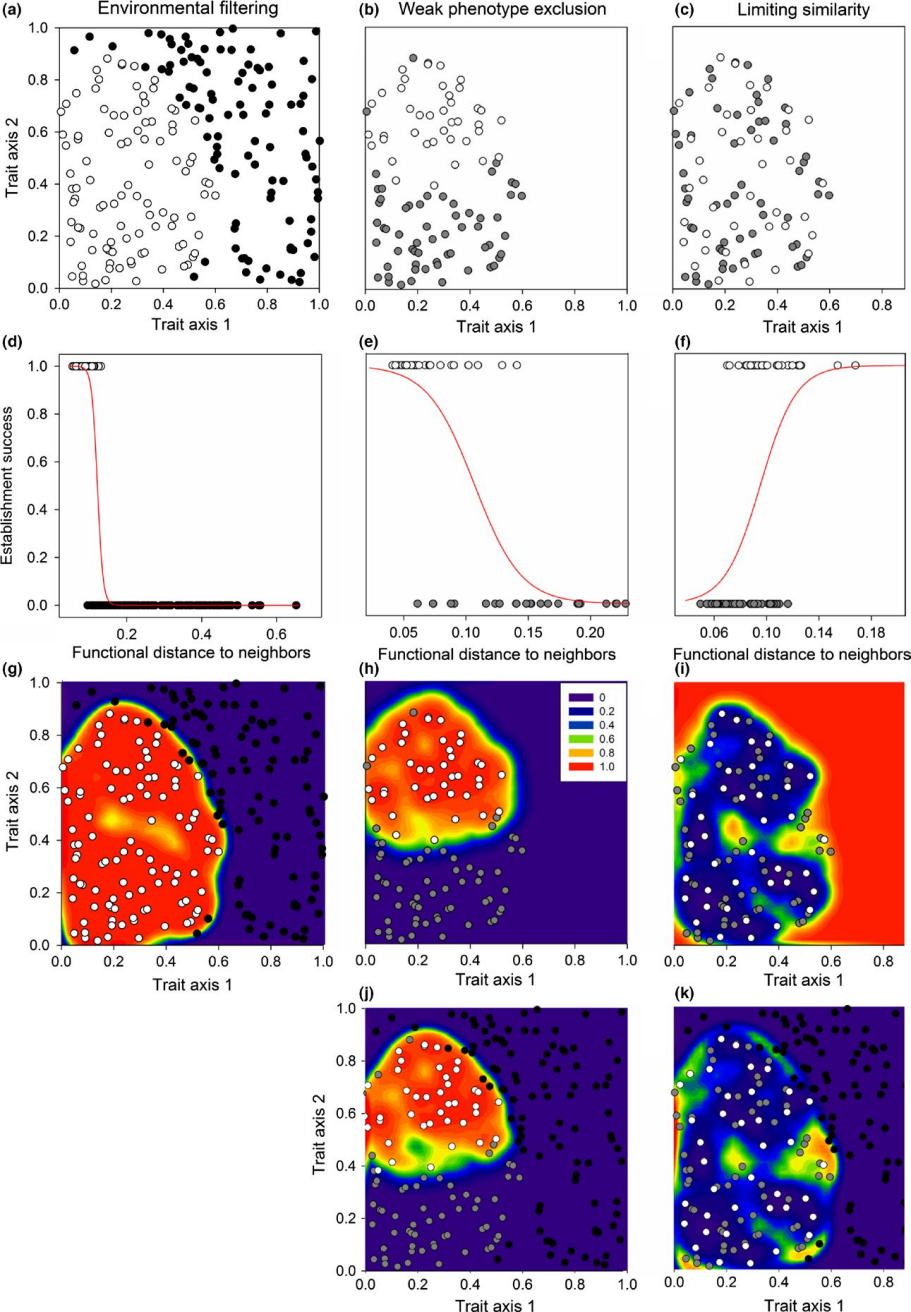
Simulated data used to illustrate how the proposed framework models environmental filtering, weak phenotype exclusion, and limiting similarity using functional dissimilarity within a hypothetical community. (a) Species that can colonize a given habitat (habitat‐specific species pool; white circles) are clustered in functional space relative to species environmentally excluded from the focal habitat (black circles). (d) To quantify the probability of being environmentally excluded, we calculated the functional distances among species pool members (white circles) and between environmentally excluded species and species pool members (black circles). Here, distances are calculated as multivariate functional neighborhood distances. We classified species within the habitat‐specific pool as successes and environmentally excluded species as failures and used logistic regression to predict the probability of other species passing the filters using these distances. (g) As expected, these regression models predict a high probability of passing the filters for species similar to the species pool, decreasing with the functional distance from the habitat‐specific species pool, and falling to zero beyond a threshold (see legend in panel h). For biotic resistance predictions (second and third columns), species from the habitat‐specific species pool are either biotically excluded (gray circles) or present locally (white circles). (b) Under weak phenotype exclusion, species within the community are clustered in functional space; (c) under limiting similarity, species within the community are dispersed. Logistic regression showed (e) a negative relationship between functional distance and establishment under weak phenotype exclusion and (f) a positive relationship under limiting similarity. Success is (h) high near locally observed species for weak phenotype exclusion, but (i) low under limiting similarity. This pattern is maintained when combined with (g) environmental filtering for (j) weak phenotype exclusion and (k) limiting similarity, except bounds on invasible areas are set by environmentally excluded species

Species that can pass environmental filters will typically possess a certain suite of traits and be clustered in trait or phylogenetic space (Figure 1a; Cornwell, Schwilk, & Ackerly, 2006; Webb et al., 2002). From the regional species list, the presence or absence of species in the habitat‐specific species pool can indicate which sets of characteristics allow species to colonize the habitat and those that are excluded through environmental filtering. The functional distances are then used in a logistic regression to estimate the relationship between dissimilarity and environmental inclusion. Within this logistic modeling framework, a separate equation is used for each habitat, although multiple habitats could be included in the model by including habitat identity in the model. Within the model, each species serves as a data point, with their presence or absence in the habitat‐specific species pool as the binary response variable. In this model, environmentally excluded species represent failures and species from the habitat‐specific species pool are successes. The functional or phylogenetic distances of these species to the habitat‐specific species pool are used as the predictor of presence or absence. To model environmental inclusion and exclusion for the hypothetical data shown in Figure 1a, we used multivariate trait distances as the predictor (Figure 1d). However, as this is a logistic regression framework, any measure of dissimilarity or distance could be used, including phylogenetic distances, individual traits, and their interactions (see Section 3 for details). After developing the logistic model, we measured the mean pairwise functional distance between each potential colonist and the species in the habitat‐specific pool. The functional distances for each colonist are then used in the logistic regression equation to estimate the probability that they will pass the environmental filters. In cases where there are multiple communities within the habitat, the same equation is used for each community. For the region shown in Figure 1a, colonists similar to the habitat‐specific pool were more likely to be successful (Figure 1g).

### 2.3 Biotic filtering

Within the habitat‐specific pool, trait dissimilarity between species observed within the community and those that are absent are used to evaluate biotic filters. If the community is structured by interactions with a dominant competitive phenotype and all inferior phenotypes are excluded (weak phenotype exclusion; Grime, 2006), species coexisting within the community should be more similar to each other than they are to biotically excluded species (Figure 1b). If similar species are unable to coexist due to strong competition for the same resources (limiting similarity; MacArthur & Levins, 1967), species coexisting within the community should be more functionally dissimilar from each other than they are from biotically excluded species (Figure 1c). Consequently, we can measure the functional distances among the species within the community and between species within the community and biotically excluded species to estimate the probability of passing the biotic filters. In this case, distances among locally observed species act as indicators of success and distances between observed and biotically excluded species act as indicators of failure (Figure 1e,f). These distances are then used in a logistic modeling framework like that used for environmental filtering, except the model only includes species within the habitat‐specific species pool as data points. In our example (Figure 1), for weak phenotype exclusion, potential colonists similar to the species within the community were more likely to establish (Figure 1e,h). In contrast, for limiting similarity, colonists dissimilar from species within the community were more likely to establish (Figure 1f,i).

After obtaining the predictions from both the environmental and biotic filtering models, we calculated the overall probability of establishment by multiplying the probabilities of passing the environmental and biotic filters. Using the same community as used throughout these examples (Figure 1), we found a tight clustering of successful phenotypes around the observed species when the community was structured by weak phenotype exclusion (Figure 1j). Conversely, when limiting similarity among the most similar pairs of species structured the community, the probability of successful establishment was highest in empty niche spaces both within the bounds of functional space occupied by observed species and along the margins of the habitat‐specific species pool (Figure 1k).

In the example shown in Figure 1, we estimated potential biotic filtering using a single community and ignored species’ abundances to keep the example simple. However, biotic interactions occur at smaller spatial scales than the whole community (Huston, 1999) and abundance can be important in determining interaction outcomes (Hillebrand, Bennett, & Cadotte, 2008). Consequently, multiple samples from the community, each containing distinct information on species abundances, may be more useful in practice. Multiple samples can easily be included in the model by measuring distances within each community sample and including community sample as a factor. Species abundances can also be included by weighting the functional distances to each species in the community by the relative abundance of that species. More details on these methods can be found in the section on applying the framework.

## 3 Measuring Dissimilarity

### 3.1 Functional neighborhood distances

**Figure 2:**
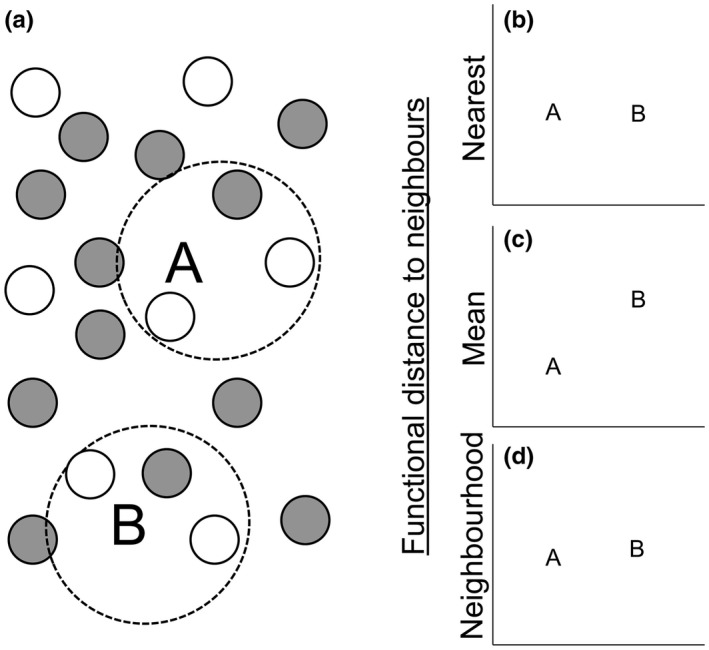
A hypothetical example showing how different functional distance measures may affect our interpretation of establishment probabilities. Panel (a) shows the distribution of species in functional space, where gray circles represent biotically excluded species and white circles species present in the community. The letters A and B represent two potential colonists. Both species are similarly distant from their nearest neighbor (b). As species A is closer to the mean trait value for the observed community than species B, species B has a higher mean distance to species within the community than A (c). Using the mean distance to species within the functional neighborhood (dashed circles), there is little difference between species A and B (d)

Nearest neighbor and mean pairwise distances are the most common distance metrics used when detecting patterns in traits and phylogenetic relationships, yet they both may be disadvantageous under certain situations. For example, in a community structured by limiting similarity, two potential colonists may be equally dissimilar to their nearest neighbors in functional space, but vary in their distance to the community mean trait value (Figure 2a). Nearest neighbor distances predict that the two species have an equal probability of establishment (Figure 2b). By contrast, mean pairwise distances predict that species closer to the community mean have a lower establishment probability (Figure 2a,c). Consequently, in this scenario, mean pairwise distances can only accurately predict establishment if establishment is driven by a species having lower or higher trait values than the resident community (e.g., taller or less palatable). Nearest neighbor distances are more likely to distinguish limiting similarity, if interactions are primarily with a single resident species. If more than one species exerts competitive pressure under limiting similarity, then more than one species should be included in the distance measurement. By calculating the distance to a subset of the species in the community (functional neighborhood distances), any number of scenarios can be accounted for. Here, we show neighborhood distances as the average to the two nearest neighbors (Figure 2a,d). However, appropriate neighborhood sizes can be estimated by sequentially increasing the subset of the community included to maximize the detection of patterns in the data. Similar procedures are used in spatial analyses to detect appropriate scales (Perry, Miller, & Enright, 2006).

**Figure 3:**
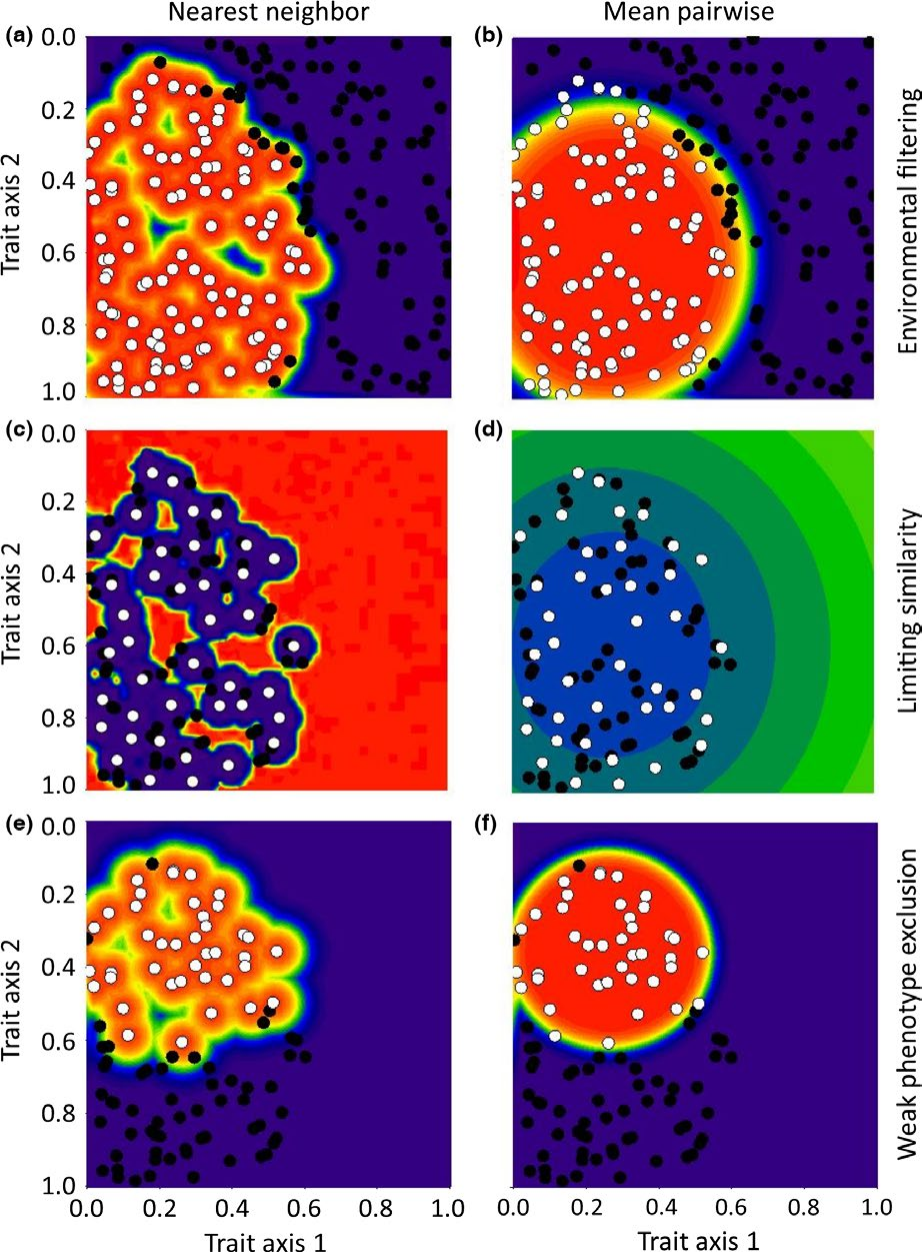
Simulated data used to show the effect of using nearest neighbor distances (left column) and mean pairwise distances (right column) on predictions of environmental filtering (top row), limiting similarity (middle row), and weak phenotype exclusion (bottom row) within a hypothetical community. These examples can be compared to Figure 1, which used the same data, but used functional neighborhood distances to calculate dissimilarity. White circles represent species that have passed the environmental or biotic filters, and black circles represent species that were excluded by that filter. The predicted probability of invasion increases with the warmth of the color (low = purple/blue, high = red/orange)

Additional behaviors of nearest neighbor, mean pairwise, and functional neighborhood distances can be identified by comparing their predictions. We do this using the same hypothetical data as used in Figure 1. When we applied the framework using nearest neighbor distances, regions of low establishment probability were detected within the cluster of species representing the effects of environmental filtering (Figure 3a) and weak phenotype exclusion (Figure 3e). Nearest neighbor distances could detect areas within the habitat‐specific species pool that were underutilized by the observed species when limiting similarity drives community assembly (Figure 3c). However, these regions overlapped the areas where environmental filtering is predicted (Figure 3a). Combined, this suggests that nearest neighbor distances may underestimate establishment in most situations. When we applied the framework using mean pairwise distances, we accurately detected the clusters of species for both environmental filtering (Figure 3b) and weak phenotype exclusion (Figure 3f), but the model did not converge when limiting similarity among the most similar pairs of species drove biotic filtering (Figure 3d). By comparison, when we used functional neighborhood distances in the framework (Figure 1, see Appendix S1 for details), the framework identified both clusters and unoccupied regions within functional space as areas of potentially successful establishment (Figure 1j,k). This suggests that functional neighborhood distances perform as a hybrid between nearest neighbor and mean pairwise distances.

### 3.2 Multivariate trait, phylogenetic, and individual trait approaches

In Figures 1 and 3, we used multivariate trait distances to demonstrate the framework. However, if limiting similarity acts on some traits and weak phenotype exclusion acts on others, patterns can be obscured using a multivariate approach (Adler, Fajardo, Kleinhesselink, & Kraft, 2013; Herben & Goldberg, 2014; Küster et al., 2008; Spasojevic & Suding, 2012). A similar disadvantage exists for phylogenetic approaches, as phylogenetic relatedness represents similarity in all conserved traits. Using phylogenetic relatedness can be advantageous if some conserved traits are unknown or not easily measured, but it also requires that the important traits are conserved and that the function of these traits is not significantly altered by other traits unrelated to the process of interest (Gerhold, Cahill, Winter, Bartish, & Prinzing, 2015; Thuiller et al., 2010). Using individual traits can overcome some of these issues, providing that the correct traits are chosen.

**Figure 4:**
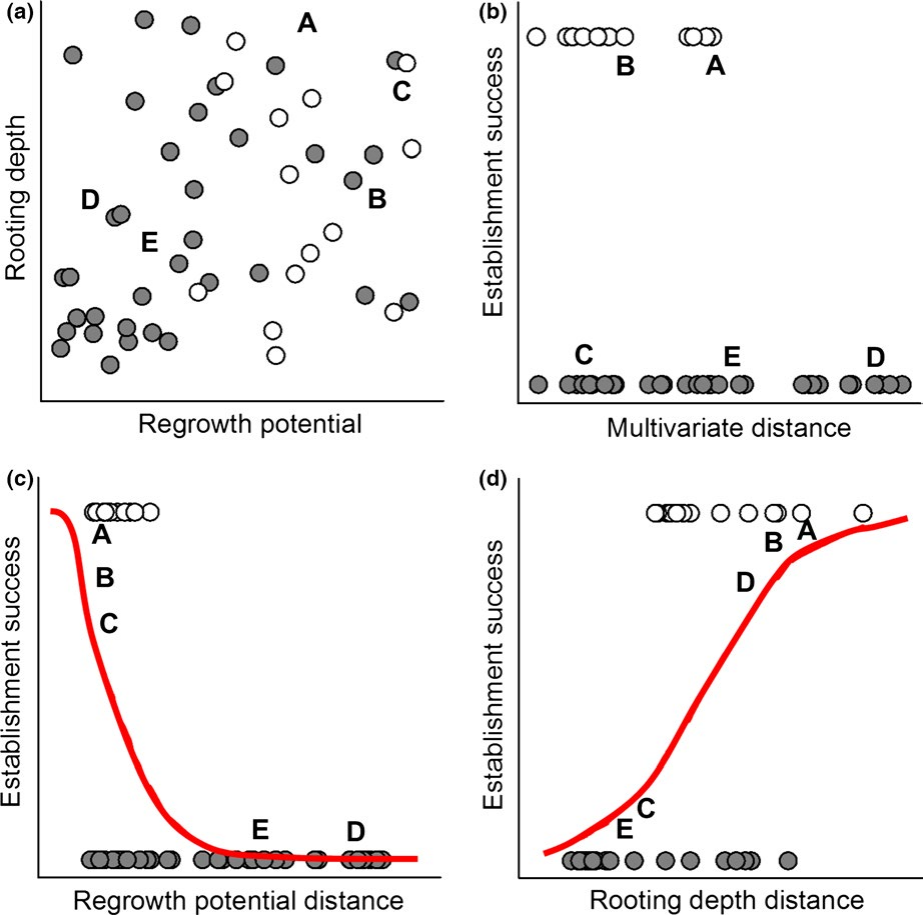
A hypothetical example using simulated data to show the differences between multivariate and individual trait approaches to predicting establishment within a single community. White circles represent species present in the community, gray circles species excluded through biotic interactions, and letters different potential colonists. Traits are randomly generated to represent regrowth potential which represents response to herbivory, and rooting depth which represents water acquisition strategies. Here, species within the community exhibit similar traits relating to herbivory tolerance (a, c), but segregate themselves according to water uptake strategies (a, d). Multivariate analyses are unlikely to detect limiting similarity in this scenario (b). Separately analyzing herbivory tolerance (c, e) and water acquisition strategy (d, f) makes the patterns easier to discern (e, f). Species A and B are likely to establish as they root at different depths than the species already within the community and have high herbivory tolerance. The other species are likely to fail: species C has no available water niche, species D cannot tolerate herbivory, and species E does not possess either required characteristic

The benefit of using an individual trait approach can be demonstrated using a hypothetical community that is structured by both herbivory and competition for water, with the two processes acting on distinct traits. If herbivory tolerance is required for persistence, species in the community should be similar in related traits (e.g., regrowth capacity) and the trait values will be clustered relative to absent species (Figure 4a). If differentiation in water acquisition is also important, coexisting species should be dissimilar in related traits (e.g., rooting depth) and the trait values dispersed (Figure 4a). As such, colonists with high regrowth potential and a dissimilar rooting depth are most likely to establish, while colonists with only one of these characteristics or neither characteristic are far less likely to establish (Figure 4). In this scenario, multivariate Euclidean distances that combine both traits did not effectively predict establishment (Figure 4b). Similarly, if both traits are conserved, phylogenetic approaches would be unlikely to detect any pattern. However, by using the individual traits as independent predictors in the model, we detected the pattern in both traits (Figure 4c,d), indicating that an individual trait approach may be most appropriate.

**Figure 5:**
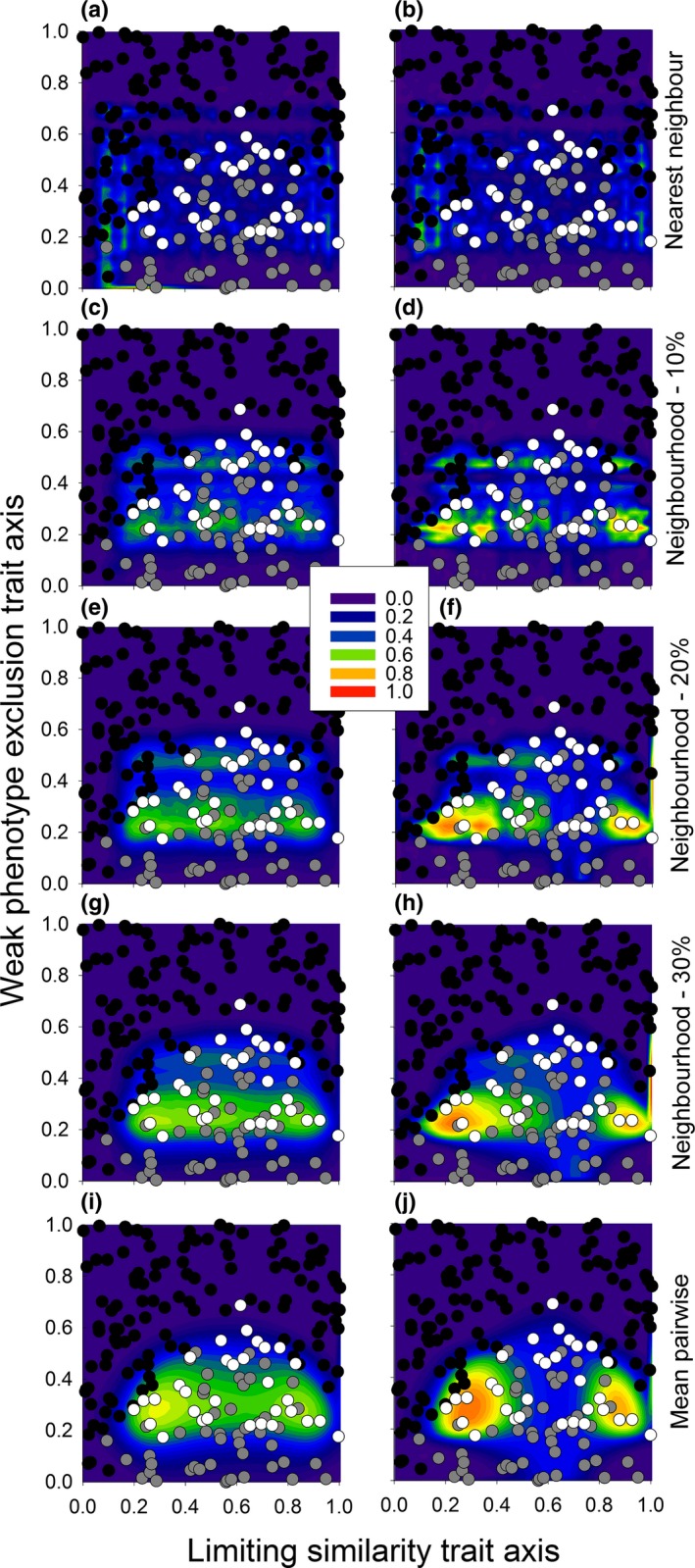
An example of the effect of neighborhood size on establishment predictions when multiple mechanisms affect community assembly. Neighborhood sizes are shown as a proportion of the total community. Figures show the effect of different functional neighborhood sizes on establishment predictions for a single community, both without (left column; a,c,e,g,i) and with (right column; b,d,f,h,j) trait interactions included in the model. The neighborhood sizes shown range from nearest neighbor distances (one species; a, b) to mean pairwise distances (all species; i, j). Red areas denote areas with high predicted establishment and purple areas low establishment (see legend between panels c‐f). In all panels, black circles denote environmentally excluded species, gray circles biotically excluded species, and white circles species observed within the community

To illustrate how interactions among traits and different neighborhood sizes affect establishment predictions using an individual trait approach, we construct another hypothetical community. For this community, species possess two randomly generated traits. Both traits are affected by environmental filtering, but for biotic filtering one trait is affected by limiting similarity and the other by weak phenotype exclusion (see Appendix S1 for details). Consequently, species from the habitat‐specific species pool are clustered in both trait dimensions, and successful establishment should occur in zones within the functional space occupied by the habitat‐specific species pool. Trait interactions appear to have little effect on environmental filtering predictions. However, the use of nearest neighbor distances mistakenly predicted success for species without the traits required to pass the environmental filters (warmer colors in areas with no species from the habitat‐specific species pool in Figure 5a,b). As we increased neighborhood sizes, this became less of an issue as successful phenotypes became restricted to the functional space occupied by the habitat‐specific species pool (Figure 5).

Within the habitat‐specific species pool, species within the community are dispersed along the x‐axis and clustered along the y‐axis (Figure 5), reflecting our simulation of limiting similarity and weak phenotype exclusion (see Appendix S1). We expect species to successfully establish if they are dissimilar to locally observed species in the trait under limiting similarity, but similar in the trait under weak phenotype exclusion. Without the inclusion of trait interactions in the model (Figure 5 left column), predicted establishment probabilities remained low irrespective of neighborhood size. With trait interactions (Figure 5 right column), the predicted probabilities increased. Focusing only on models with trait interactions, using nearest neighbor distances did not predict successful establishment in any area of functional space (Figure 5b). Using intermediate neighborhood sizes detected distinct combinations of functional traits that should lead to successful establishment for the trait affected by limiting similarity (Figure 5d,f,g). For the trait affected by weak phenotype exclusion, we detected multiple clusters of traits when using intermediate neighborhood sizes. These clusters were present in the data, although they were not programmed into the example. Interestingly, these clusters were not detected by mean pairwise distances. Mean pairwise distances also only predicted success for extreme trait values, relative to the habitat‐specific species pool, when the trait was affected by limiting similarity (Figure 5h,j). Combined, these patterns suggest that trait interactions are necessary to detect the signature of multiple assembly processes using the current framework. The most appropriate neighborhood size for detecting these processes will depend on the precise trait patterns within the habitat‐specific species pool and the community. However, nearest neighbor distances functioned poorly when detecting environmental filtering or weak phenotype exclusion and mean pairwise distances only predicted successful establishment along the boundary of the habitat‐specific pool when detecting limiting similarity. Only intermediate neighborhood sizes detected both trait clustering and empty niches within the habitat‐specific species pool. They were also the only distance measure to detect multiple distinct trait clusters for the trait affected by limiting similarity.

## 4 Applying the Framework: Predicting Plant Species Establishment in Calcareous Grasslands

To demonstrate the application of the modeling framework and test the accuracy of its predictions, we used data from two highly similar seed addition experiments located in calcareous grassland sites in Estonia (Zobel et al., 2000, 2005). Importantly, these grasslands are highly studied ecosystems; as such both plant occupancy and trait data were available.

### 4.1 Experimental design

Both experimental sites are located within 10 km of each other near Virtsu, Estonia (58°34′12″N, 23°31′26″E). One site is an alvar grassland with a shallow humus layer (10–15 cm), 170 g/m^2^ live biomass, and 13 species per 0.01 m^2^ (Zobel et al., 2000), while the other is a more productive and diverse wooded meadow with a deeper humus layer (20–25 cm), standing biomass of 250 g/m^2^, and 16 species per 0.01 m^2^ (Zobel et al., 2005). More thorough site descriptions can be found in the original publications (Zobel et al., 2000, 2005).

At both sites, a series of 10 × 10 cm plots were established, 60 in the alvar and 40 in the meadow. Seeds of multiple herbaceous species were added to half the plots at each site. At the alvar, 15 species were added, all of which were either absent or uncommon at the focal site, but native to Estonian alvar grasslands (Zobel et al., 2000). In the meadow, 25 species were added: 14 native to Estonia, but locally absent, and 11 alien to Estonia. The alien species were all Eurasian and able to reproduce in the Estonian climate, but not classified as invasive (Zobel et al., 2005). Native seeds were collected from surrounding areas and exotic seeds from plants growing at the University of Tartu Botanical Garden. For the alvar study, 15 seeds were added to each plot per species, whereas for the meadow, seed addition rates varied between 5 and 20 seeds per species per plot, with more seeds added for species with smaller seeds. In each study, the number and identity of all individuals in each 10 × 10 cm plot were recorded monthly during the growing season for three years following seed addition; however, we only use the final estimates of species composition and abundance.

### 4.2 Constructing regional species lists and habitat‐specific pools

We constructed regional lists and habitat‐specific species pools for each site separately, focusing on herbaceous species for the latter. Regional species lists were developed from the Atlas of Estonian Flora (Kukk & Kull, 2005) and included all species within a 10 × 10 km grid cell containing the study location. Habitat‐specific species pools were constructed using species lists from the target site and similar habitats within the same county (Läänemaa): 7 sites for the alvar and 4 sites for the meadow (Kukk & Elvisto, 2013; Pärtel, Mändla, & Zobel, 1999). However, to avoid including species that were potentially misidentified or that were not usually found in these habitat types, we excluded all species that were only found in one site per habitat type when constructing the habitat‐specific species pool. We also excluded all species from the additional sites that were not recorded within the 10 × 10 km grid that we used to construct the regional species list as those species may have been unable to disperse to the focal site. The resultant regional species lists contained 489 species for the alvar and 590 species for the meadow, whereas the habitat‐specific species pools contained 87 species for the alvar and 228 species for the meadow.

### 4.3 Trait data

Trait data were gathered from publicly available trait databases. Height, specific leaf area (SLA), and seed weight were included as they are important indicators of plant strategies (Westoby 1998) and were readily available from LEDA (Kleyer et al. 2008), EcoFlora (Fitter & Peat 1994), the Seed Information Database (Royal Botanic Gardens Kew 2014), or other published sources (Pierce, Brusa, Vagge, & Cerabolini, 2013). For height, SLA, and seed weight, we used the average trait value from the data available and log‐transformed these values prior to analyses due to high positive skew. We also included Ellenberg numbers, which represent an ordinal classification scale of plant habitat preferences for a number of important niche axes. Ellenberg numbers were largely taken from the original classification (Ellenberg et al. 1991); however, for species that were absent from that database or for which some habitat preferences were not evaluated, missing data were taken from EcoFlora. For all analyses, we included Ellenberg numbers for soil moisture (F), soil fertility (N), light availability (L), and soil pH (R). Complete trait data were available for all 15 added species in the alvar study and for 8 native and 3 alien added species from the meadow study (see Table S1). Added species with incomplete trait data were excluded from the analysis. Complete trait data were also available for 87% of the 639 species (556 species total) in the combined regional pools. Only these species were used in model development.

### 4.4 Analyses

To model environmental filtering, we calculated the functional dissimilarity between species in the regional list and the habitat‐specific pool as Gower distances (Laliberté & Legendre, 2010). All traits were standardized to range from zero to one prior to distance calculations. Neighborhood sizes ranged from nearest neighbor distances to mean pairwise distances in 10% increments. The percentages were multiplied by the size of the habitat‐specific species pool in each study to determine the number of species in the neighborhood, always rounded up. For each neighborhood size, distances were calculated as the average among species within the neighborhood. These distances were then used as explanatory variables to model the probability of passing the environmental filters using logistic regression in R, with habitat‐specific pool species as indicators of success and environmentally excluded species as indicators of failure. Individual traits were treated as separate variables, with separate models run for each site.

Biotic filtering was modeled similarly to environmental filtering, with some important differences. We used the 10 × 10 cm plots as community samples and calculated trait distances relative to the remainder of the habitat‐specific pool within each sample. All distances were abundance weighted by multiplying the distances by the proportion of individuals belonging to the observed species within the plot, with the total of these weights summed to one for each plot. However, the distance matrix was centered first, so that when weighting by abundances, highly similar or dissimilar species could have equal weights, but with opposite signs. Inclusion in the neighborhood was determined using abundance weighted distances and the same range of proportional neighborhood sizes as environmental filtering. With these data, we constructed binomially distributed generalized mixed models for each site using the R package lme4 (Bates et al. 2014). Each model used presence or absence in the observed community as the dependent variable, neighborhood distances for each individual trait as fixed factors, and plot identity as a random factor.

For both environmental and biotic filtering, we tested the effect of trait interactions on model fit and establishment predictions. We compared three sets of models: (1) models with no trait interactions; (2) models with all pairwise interactions; and (3) models where interaction terms were dropped to minimize AICc (the most parsimonious models). However, for the environmental filtering models, interactions among ordinal traits were excluded as there was insufficient variation among species when using smaller neighborhood sizes (≤10%), resulting in models that did not converge.

To predict establishment for the focal species, we used their functional neighborhood distances to the habitat‐specific species pool and the species observed in each plot in the corresponding environmental and biotic models. For the biotic models, we used the average of the probabilities across community samples as the estimated probability of passing the biotic filters for each species. We then calculated the overall probability of establishment by multiplying the environmental and average biotic probabilities. We calculated these overall probabilities for all combinations of neighborhood sizes between the environmental and biotic models.

To test the accuracy of the predictions, we compared the estimated overall probability of establishment with actual establishment. We calculated actual establishment as the average proportion of seeds that established for each focal species (plants in year three/seeds added) across all plots. We then used a linear model with actual establishment as the response variable and predicted establishment as the explanatory variable. To account for differences among sites, we also included site as a factor in the model. Initially, we ran the model including all species. We then repeated the analysis with only native species (23 of 26 species). We repeated this procedure for all environmental and biotic neighborhood size combinations.

## 5 Results and Discussion

**Figure 6:**
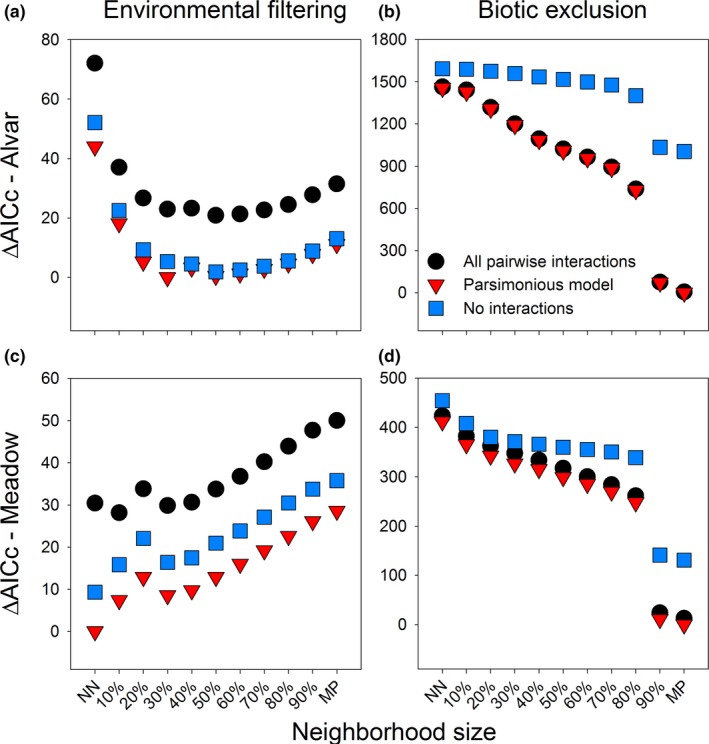
The effect of different functional neighborhood sizes on the fit of models describing environmental (left) and biotic filtering (right) at the alvar (top) and meadow sites (bottom). Relative model fit was measured as the change in AICc from the best‐fitting model. Shown are models with no interaction terms among traits (blue squares), models with all pairwise interactions among traits (black circles), or the most parsimonious model (red triangles)

The functional neighborhood sizes that best described environmental and biotic filtering were similar between the two sites, with some caveats. At the alvar, neighborhood sizes ranging from 20% to 90% performed similarly when describing environmental filtering, with 30% performing best (Figure 6a), whereas at the meadow, model fit decreased with neighborhood size more dramatically (Figure 6c). Nearest neighbor distances had the highest AICc score at the alvar, but the lowest at the meadow when modeling environmental filtering. However, at both sites, nearest neighbor distances resulted in parameter estimates orders of magnitude greater than those seen for other neighborhood sizes and these estimates were mostly nonsignificant (*p* > .95, Table 1). This indicates that nearest neighbor distances poorly described the underlying trait patterns, despite low AIC scores. These unrealistic parameter estimates may have resulted from limited variation among species in neighborhood distances for ordinal traits when using smaller neighborhood sizes (nearest neighbor or 10%; Figure S1). As neighborhood sizes increased, the amount of variability in trait distances increased (Figure S1). Consequently, the 30% neighborhood size, despite being the third best‐fitting model for the meadow site, exhibited greater variation in trait distances and significant parameter estimates and was selected as the best model (Table 1; Figure S1). These results caution against selecting a model based purely on AIC. At a minimum, the distribution of distances and the resulting parameter estimates should be examined.

**Table 1:** Standardized parameter estimates for environmental and biotic filtering models

Factor	Alvar environ. filtering			Alvar biotic filtering			Meadow environ. filtering			Meadow biotic filtering		
	NN	30%	MP	NN	30%	MP	NN	30%	MP	NN	30%	MP
Moisture	0.75	−**1.35**	−**0.78**	**0.37**	**0.41**	**1.72**	−6.26			−**0.28**	−**0.33**	−**0.46**
Light	−9.35	−**0.66**	−**0.59**	**0.10**	**0.16**	**0.42**	−184.71	−**0.44**	−**0.42**	0.08	**0.15**	**0.21**
Fertility	−2.87	−**0.82**	−**0.76**	0.06	**0.24**	**0.75**	−8.72	−**1.03**	−**0.62**	0.05	0.06	0.09
pH	−6.88		−0.20	−**0.10**	**0.31**	**1.22**	−4.95	−**0.20**	−0.07	**0.14**	**0.14**	**0.28**
SLA	−**0.32**	−**0.95**	−0.41	**0.14**	0.07	**0.17**	−104.43	−**0.56**	−**0.31**	0.07	0.08	0.10
Height	1.10		0.05	0.08	**0.27**	**1.03**	−**3.51**	−**1.54**	−**1.05**	**0.31**	**0.38**	**0.57**
Seed weight	−0.46	−0.32	−0.36	−**0.11**	−**0.28**	−**0.61**		−0.09	−0.10	−**0.35**	−**0.46**	−**0.61**
Moisture:light				−**0.12**	−**0.41**	−**1.00**						
Moisture:fertility				**0.18**		−**0.50**				−0.06		0.10
Moisture:pH				0.07								**0.13**
Moisture:SLA		−1.18		**0.08**	**0.51**	**0.88**					−**0.11**	−**0.29**
Moisture:height	12.62				−**0.15**	−**0.99**					−**0.11**	−**0.28**
Moisture:seed		0.33	0.36		−**0.11**	−**0.29**				−**0.15**	−**0.19**	−**0.36**
Light:fertility				−0.06		**0.15**				**0.07**		
Light:pH				−0.06						**0.08**		
Light:SLA				**0.14**		−**0.30**	−691.22			−**0.12**	−**0.15**	−**0.35**
Light:height				−**0.28**	−**0.25**	−**0.45**				**0.07**		−0.05
Light:seed				**0.09**	**0.13**			−0.44	−0.26	**0.07**	**0.13**	**0.32**
Fertility:pH				−0.08	**0.15**	**0.49**				**0.07**	0.08	
Fertility:SLA					−**0.51**	−**0.63**						
Fertility:height				−**0.10**		−**0.49**						−**0.09**
Fertility:seed					−**0.10**	**0.29**				−0.07		
pH:SLA			−0.5	**0.14**	**0.29**	**0.34**		−0.28	−0.23			**0.17**
pH:height						**0.51**				−**0.12**		**0.09**
pH:seed					**0.22**	**0.26**						**0.17**
SLA:height	**0.82**		**0.27**					−0.84				**0.14**
SLA:seed				−**0.26**	−**0.42**	−**0.42**						
Height:seed				**0.11**	**0.13**	−**0.56**		−0.99	−**0.74**			

NN, Nearest neighbor; MP, mean pairwise.

Parameters in bold are significant at *p* < .05. Empty cells mean the parameter was excluded during model selection.

We expected larger neighborhood sizes to better explain environmental filtering due to their increased power to detect clusters of successful species (Figures 3b,f and 5j), but they poorly explained environmental filtering in our data (Figure 6a,c). In the example shown in Figure 5, intermediate neighborhood sizes detected multiple trait clusters in relation to weak phenotype exclusion, seen as multiple hotspots of establishment surrounding observed species (Figure 5d,f,h). Consequently, the better fit for intermediate neighborhood sizes may indicate the presence of multiple trait clusters within the habitat‐specific species pools for these two sites and consequently multiple strategies or functional groups (Cadotte, Cavender‐Bares, Tilman, & Oakley, 2009; Reich, 2014).

For both the alvar and the meadow sites, biotic filtering was best modeled using mean pairwise distances (Figure 6b,d), consistent with previous work (Carboni et al., 2016; Gallien et al., 2014). The models also detected both significant clustering and dispersion among species within the communities (Table 1). In the example illustrated in Figure 5, mean pairwise distances were always the best at detecting patterns of trait clustering within the community. However, for traits affected by limiting similarity in this same example, the use of mean pairwise distances predicted that only species with high or low values for that trait would be successful (Figure 5j). Many processes may lead to such a pattern. Here, for example, species were dispersed in height, SLA, and light preferences at both sites (Table 1). This suggests that species may vertically partition space resulting in clusters of tall, fast‐growing species that require a large amount of light and of short, slower‐growing species that are shade tolerant. Other mechanisms may explain the remaining trait patterns, but a full exploration of these relationships is beyond the scope of this paper. However, it is noteworthy that including interactions among traits always improved model fit (Figure 6), highlighting the importance of trait interactions in species establishment (Küster et al., 2008).

**Figure 7:**
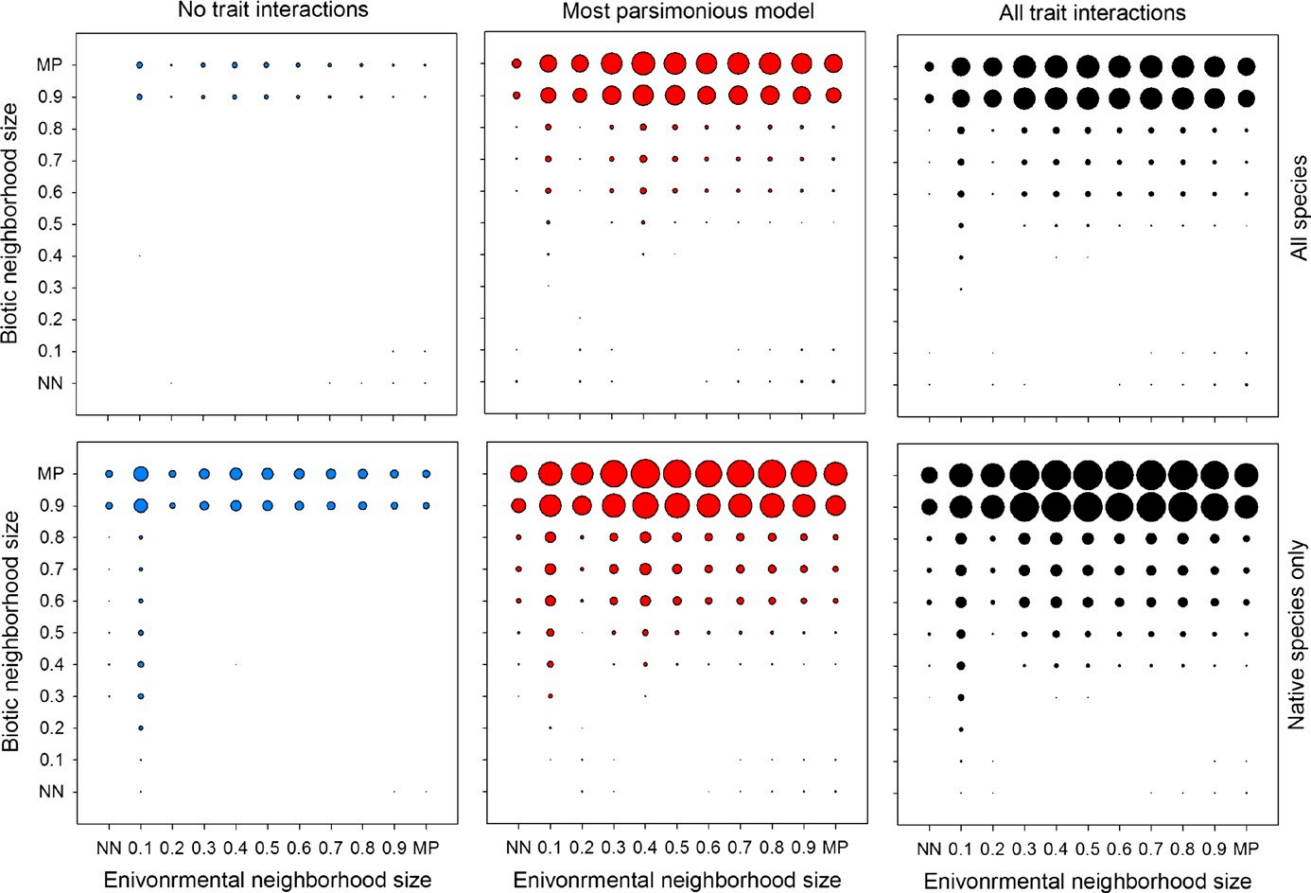
The variation in actual establishment explained by model predictions for species added to grassland sites as seed. The diameter of the circles is proportional to the variation explained (adjusted *R* ^2^), with a maximum value of .43 with all species and .54 with only native species. Predictions are from all possible combinations of neighborhood sizes used for modeling environmental and biotic filtering. Shown are predictions with no interactions (left), the most parsimonious models (middle), models with all pairwise interactions among traits (right), both with (top) and without (bottom) alien species

Overall, the model‐based predictions of establishment matched actual establishment well; however, the fit was dependent on the inclusion of interaction terms and selection of neighborhood size (Figure 7). Including interaction terms greatly improved the accuracy of model predictions (Figure 7). The predictions using the full models explained more variation in establishment than the most parsimonious models, although this difference was small (Figure 7). Regardless of whether interaction terms were included, focusing on only added native species increased model fit by 33% on average. The effects of different neighborhood sizes were also very pronounced. In the models of environmental filtering, neighborhood sizes ranging from 30% to 90% all did reasonable jobs of predicting establishment, whereas for biotic filtering, neighborhood sizes of 90% or 100% were the only neighborhoods that successfully predicted establishment. The large differences between these neighborhood sizes and all smaller neighborhood sizes highlight the importance of including most species when assessing the effects of biotic interactions at this spatial scale in these communities. Importantly, these results were consistent with the two neighborhood sizes identified as fitting the data the best during model development (30% and mean pairwise for environmental and biotic filtering, respectively). This indicates that selecting an appropriate neighborhood size using model selection and parameter examination can be meaningfully used to predict establishment.

**Figure 8:**
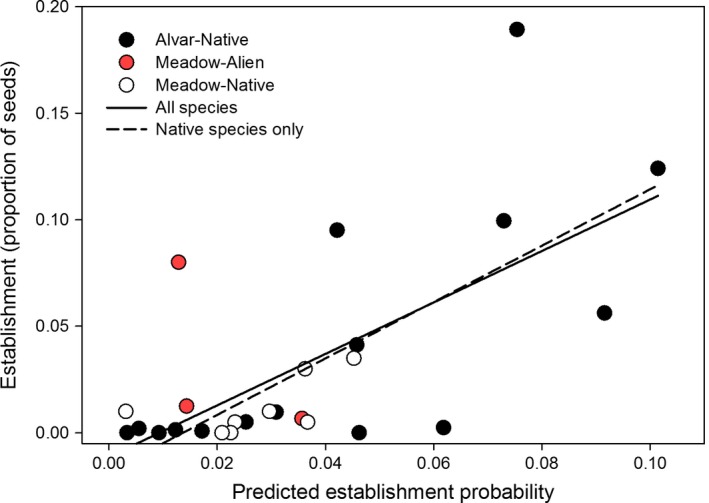
Predicted establishment versus actual establishment from seed using predictions from the best‐fitting neighborhood sizes (Figure 6). Native alvar species are shown in black, native meadow species in white, and alien species added to the meadow in red. Fitted models included site and predicted establishment as fixed effects, with separate models run for all species (solid line) and only native species (dashed line)

Focusing only on the neighborhood sizes selected using our modeling procedure, our predictions of establishment potential explained between 40 and 50% of the variation in actual establishment. When we included all species in the model, predicted establishment was highly significant (*t* = 3.98, *p* < .001) with no differences between sites (*t* = −0.23, *p* = .816) and an adjusted *R* ^2^ of .40 (Figure 8). We found similar results when using only native species (predicted establishment *t* = 4.48, *p* < .001; site *t* = −0.63, *p* = .539) with a large increase in model fit (adj. *R* ^2^ = .50; Figure 8). This difference in model fit was likely driven by the removal of a single outlying alien species (Figure 8). Nevertheless, the strong relationships between predicted and actual establishment indicates high potential for predicting establishment using the proposed framework. Whether this framework also applies to alien species is unclear as the small number of alien species included does not allow a thorough evaluation. The applicability of assembly rules to the establishment of alien species may also depend on the similarities between alien and native species, which remains a contentious issue (e.g., Dawson, Maurel, & van Kleunen, 2015; Leffler, James, Monaco, & Sheley, 2014, 2015).

## 6 Conclusions

The likelihood that a species will establish within a given community depends on their dissimilarity to the resident community and the processes that structure the community (Laughlin et al., 2012; MacDougall et al., 2009; Shipley et al., 2006). Including information on absent species has improved our understanding of community assembly (de Bello et al., 2012; Chalmandrier et al., 2013) and can improve predictions of which species will establish. The accuracy of such predictions, however, is highly dependent on how dissimilarity is measured. The proposed framework can identify appropriate functional neighborhood sizes for measuring dissimilarity. Also, given the strong effect of trait interactions on species establishment both here and in other studies (Küster et al., 2008), we strongly suggest that future work include such interactions. By doing so, we greatly improve our ability to understand and predict community assembly.

## Conflict of Interest

None declared.
