# Supplementary material for: Predicting species establishment using absent species and functional neighborhoods
Source: Ecol Evol. 2017 Mar 4;7(7):2223–37. doi: 10.1002/ece3.2804 (PMC5383500; doi:10.1002/ece3.2804)

**Appendix S1** Additional simulation details and results.

**Invasion and functional similarity: Using absent species to improve predictions**

Jonathan A. Bennett and Meelis Pärtel

**Additional methods**

*Community simulations*

To explore the effect of neighborhood size on our ability to detect different patterns, we created random habitat-specific species pools with randomly generated trait values. We generated new species pools for each set of simulations. For each simulation we eliminated species from the species pool through either limiting similarity or weak phenotype exclusion. Limiting similarity was simulated by iteratively removing the species with the shortest distances to their neighbors until a predetermined community size was reached. In cases where species tied for the shortest distance, one species was selected randomly. For all simulations, we used nearest neighbor distances when simulating limiting similarity, following Kraft and Ackerly (2010). Weak phenotype exclusion was simulated by generating a random trait optimum and eliminating species with the greatest distance to that optimum until a specific community size was reached. When either limiting similarity or weak phenotype exclusion were acting upon multiple traits, we excluded species with either the shortest total distance for limiting similarity or greatest total distance for weak phenotype exclusion. If both limiting similarity and weak phenotype exclusion were structuring the community, we first excluded species by weak phenotype exclusion and then limiting similarity, with the proportion of species excluded by each process proportional to the number of traits affected by that process. By conducting the simulation in this way, we assume that weak phenotype exclusion acts as an initial biotic filter, following which species compete via limiting similarity to determine which species coexist.

When testing the effect of different neighborhood sizes using multivariate trait distance, we began with a regional list of 200 species with random scores for two hypothetical traits between 0 and 1. Environmental filtering was set to reduce the regional list from 200 species to 100 species that belong in the site-specific pool (Fig. 1a), with the remainder treated as being environmentally excluded. For both limiting similarity and weak phenotype exclusion, the site-specific pool was reduced until observed diversity contained 40 species, with 60 species belonging to dark diversity (Fig. 1b,c).

When testing the predictions of establishment in a community structured by environmental filtering, weak phenotype exclusion, and limiting similarity, we used a regional species list of 200 species, a habitat specific species pool of 80 species, and an observed richness of 30 species. The species each possessed two functionally important traits. For biotic filtering, one of these traits was affected by limiting similarity and the other by weak phenotype exclusion. For this community, we measured functional neighborhood distances at neighborhood sizes ranging from nearest neighbor distances to mean pairwise distances in 10% increments.

For all simulations, we used the logistic modelling framework outlined in the main text to test how neighborhood size affected the detectability of different community assembly processes, with neighborhood sizes ranging from one species (nearest neighbor) to all species (mean pairwise). For mean pairwise distances, we cannot include the distance between an observed species and itself resulting in different numbers of species in the neighborhood for observed and absent species. Consequently, we limited the neighborhood sizes of absent species to the same size as observed species. We then used these neighborhood distances in logistic regression models (using the glm function in R). For multivariate distances, we used a single predictor in the models; whereas for the individual trait models each trait was included as an independent predictor. We also repeated the logistic regression analyses for the individual trait models, but included interaction terms between the traits. In all cases, model predictions were visualized using 2,000 species with randomly generated traits. For these species, we calculated their neighborhood distances and used the equations from the models to predict their establishment. This procedure was repeated for environmental and biotic filtering for each neighborhood size.

**References**

Kraft, N.J. & Ackerly, D.D. (2010) Functional trait and phylogenetic tests of community assembly across spatial scales in an amazonian forest. *Ecological Monographs,* **80,** 401-422.

**Figure S1.** The distribution of neighborhood distances among species in the meadow regional species list for ordinal traits at different neighborhood sizes when modelling environmental filtering. Shown are Ellenberg preferences for fertility (top), moisture (second row), reaction (pH; third row), and light (bottom). Neighborhood sizes represent only one species (left), 10% of the habitat-specific species pool (center), and 30% of the habitat-specific species pool.


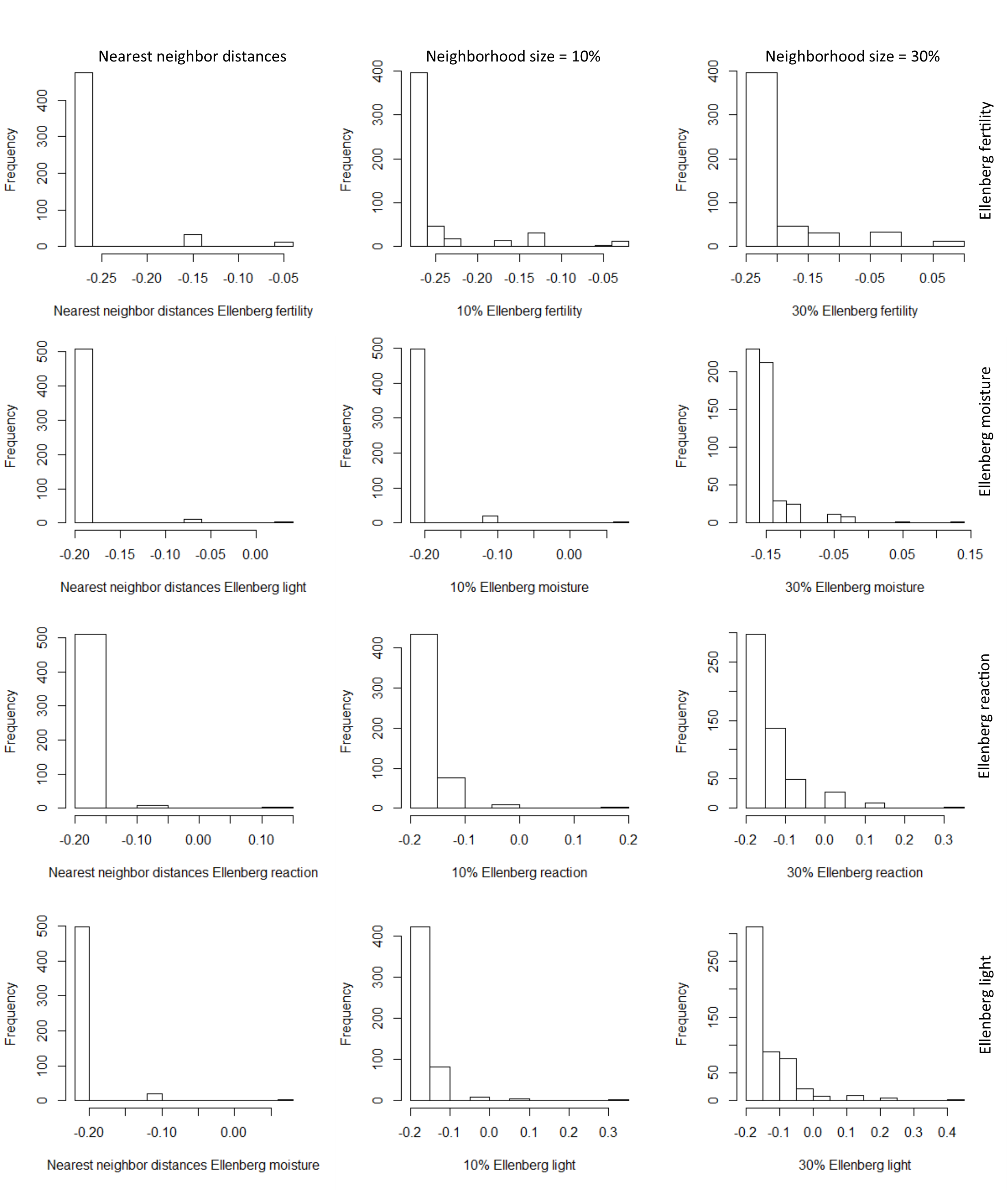

Supplement: Supplementary file 1 [file ECE3-7-2223-s001.docx]
